# TTFields alone and in combination with chemotherapeutic agents effectively reduce the viability of MDR cell sub-lines that over-express ABC transporters

**DOI:** 10.1186/1471-2407-10-229

**Published:** 2010-05-23

**Authors:** Rosa S Schneiderman, Esther Shmueli, Eilon D Kirson, Yoram Palti

**Affiliations:** 1 NovoCure Ltd., MATAM Advanced Technology Centre, Haifa 31905, Israel; 2 Rappaport Faculty of Medicine, Technion - Israel Institute of Technology, Haifa 32000, Israel

## Abstract

**Background:**

Exposure of cancer cells to chemotherapeutic agents may result in reduced sensitivity to structurally unrelated agents, a phenomenon known as multidrug resistance, MDR. The purpose of this study is to investigate cell growth inhibition of wild type and the corresponding MDR cells by Tumor Treating Fields - TTFields, a new cancer treatment modality that is free of systemic toxicity. The TTFields were applied alone and in combination with paclitaxel and doxorubicin.

**Methods:**

Three pairs of wild type/MDR cell lines, having resistivity resulting from over-expression of ABC transporters, were studied: a clonal derivative (C11) of parental Chinese hamster ovary AA8 cells and their emetine-resistant sub-line Emt^R1^; human breast cancer cells MCF-7 and their mitoxantrone-resistant sub lines MCF-7/Mx and human breast cancer cells MDA-MB-231 and their doxorubicin resistant MDA-MB-231/Dox cells. TTFields were applied for 72 hours with and without the chemotherapeutic agents. The numbers of viable cells in the treated cultures and the untreated control groups were determined using the XTT assay. Student t-test was applied to asses the significance of the differences between results obtained for each of the three cell pairs.

**Results:**

TTFields caused a similar reduction in the number of viable cells of wild type and MDR cells. Treatments by TTFields/drug combinations resulted in a similar increased reduction in cell survival of wild type and MDR cells. TTFields had no effect on intracellular doxorubicin accumulation in both wild type and MDR cells.

**Conclusions:**

The results indicate that TTFields alone and in combination with paclitaxel and doxorubicin effectively reduce the viability of both wild type and MDR cell sub-lines and thus can potentially be used as an effective treatment of drug resistant tumors.

## Background

Multidrug resistance (MDR) [1] is encountered when cancer cells are exposed to chemotherapeutic agents for a few replication cycles. It is manifested in reduced sensitivity to both the specific chemotherapy as well as to a number of structurally unrelated agents. This phenomenon obviously poses a serious impediment to successful chemotherapy. Three decades of multidrug resistance research have identified a number of mechanisms by means of which cancer cells elude the effects of chemotherapeutic agents. The most often encountered MDR is the one resulting from over-expression of ATP-binding cassette transporters such as P-glycoprotein (MDR1), multidrug resistance-associated protein-1 (MRP1), and the breast cancer resistance protein (BCRP) [1-3]. These transporters, that recognize substrates of diverse chemical nature, lower the intracellular concentration of these substrates and are normally involved in detoxification [4,5].

MDR can potentially be overcome by the use of antitumor modalities that are not involved in membrane transport, for example, anti-angiogenic agents and physical modalities such as radiotherapy, heat and electric fields. Different types of electric fields were reported to inhibit cancer cell proliferation and cause cancer cell destruction, for example: exposure of cancer cells to low amplitude DC currents [6], low intensity, low frequency (50 Hz) AC currents [7] and the intermediate frequency (100-300 kHz) alternating electric fields, termed TTFields [8-12].

TTFields are a new physical cancer treatment modality that has recently been demonstrated to be highly effective when applied to cell cultures, animal cancer models, as well as patients suffering from locally advanced and/or metastatic solid tumors [8-12]. TTFields are alternating electric fields of low intensity (1-3 V/cm) and intermediate frequency (100 - 300 kHz) that are generated by special insulated electrodes applied to the skin surface. These specially tuned fields have no effect on quiescent cells while having an anti-proliferation and destructive effect on mitotic cells. This effect is due to the fact that during cytokinesis, TTFields exert forces that move charged or polar macromolecules and organelles towards the narrow neck, separating the newly forming daughter cells [8,9]. They also interfere with the polymerization processes of the microtubule spindle during cell division. Thus, TTFields disrupt the cell structure, inhibit cell division and result in cell death. In contrast to most anti-cancer agents, TTFields are not associated with any meaningful systemic toxicity [9-12]. Furthermore, it was recently shown that TTFields may be used clinically, not only as an anti-proliferation agent, but also as effective adjuvant to currently used chemotherapeutic agents [9].

In view of the above, the target of the present study was to test the possibility of using TTFields for treating multidrug resistant cancerous and non cancerous cell lines, both as a standalone treatment and in combination with chemotherapy.

## Methods

### Materials

All cell culture media, serum and media supplements were obtained from Biological Industries, Beth Haemek, Israel. All drugs and chemical agents were obtained from Sigma.

### Cell lines

The following cell lines and their drug resistant derivatives were used: A clonal derivative (C11) of parental Chinese hamster ovary AA8 cells and their emetine-resistant sub-lines Emt^R1 ^cells having ATP dependent MDR1 type drug resistance [13], a kind gift from Prof. G. Eytan Dept. of Biology, Technion, Haifa, Israel; Human breast cancer wild type MCF-7 cells, obtained from ATCC and their mitoxantrone-resistant sub-lines MCF-7/Mx having ABCG2 transporter [14], a kind gift from Prof. M. Liscovitch, Dept. of Biological Regulation Weizmann Institute of Science, Rehovot, Israel; Human breast cancer wild type MDA-MB-231 cells obtained from ATCC and from which doxorubicin resistant MDA-MB-231/Dox cells were developed in our laboratory using a stepwise increase in drug concentration protocol. This procedure is identical with that developed for these cells in other laboratories [15] for inducing MDR1 type of ABC transporters. The AA8/Emt^R1 ^cell lines were maintained as a monolayer in -minimal essential medium containing 5% fetal calf serum, 2 mM glutamine, 100 units/ml penicillin G, and 100 μg/ml streptomycin sulphate. The Emt^R1 ^cell medium also included 1 μM of emetine. The MCF-7/MCF-7MX and MDA-MB-231/MDA-MB-231Dox cell lines were maintained under monolayer conditions in DMEM containing 10% fetal calf serum, 2 mM glutamine, 100 units/ml penicillin G, and 100 μg/ml streptomycin sulphate. The MCF-7/Mx cell medium also included 250 nM of mitoxantrone and the MDA-MB-231/Dox cells medium also included 0.1 μM of doxorubicin.

All cells were kept in a 5% CO_2 _incubator at 37°C. Exponentially growing cells were passaged twice a week using a standard trypsinization procedure.

### Cytotoxicity assay

The level of resistance to doxorubicin and paclitaxel was determined by means of the XTT assay as previously described [8,9]. Briefly, 2 × 10^4 ^cells/well were plated in 24-well plate (NUNC), incubated without drugs for 24 h and then the initial number of cells, OD_0_, was determined following incubation of with the XTT reagent using ELISA Reader (TECAN Sunrise, USA). The medium was then exchanged with ones containing different drug concentrations, 4 wells for each drug concentration (doxorubicin: 0.001-100 μM; paclitaxel: 0.0001-100 μM). After 72 h, the culture media was discharged, XTT reagent was added and the final cell number, OD_72 h_, was determined. Data obtained from 3 - 5 experiments were collected and the mean values and standard deviations (SEM) of OD_72 h_, representing final number of viable cells, were calculated for each drug concentration. Cell survival was presented as percentage of viable cells as compared to the corresponding viable cell number in no - drug controls. Drug concentrations inhibiting cell growth by 50% (IC_50_) were calculated from relative survival curves using the median-effect principle [16].

### Exposure to TTFields

As previously described [9,11], two pairs of electrodes, insulated by a ceramic having a very high dielectric constant (NovoCure Ltd, Haifa, Israel), were positioned at 90° with respect to each other in both treatment and control Petri dishes. The distance between the electrodes in each pair was 20 mm. Each pair of electrodes was alternatively connected for 250 ms to a sinusoidal waveform generator (NovoTTF, NovoCure Ltd. Haifa, Israel) that produced 1.75 V/cm, 150 kHz fields in the medium [8]. The 150 kHz frequency of TTFields was found to be effective for treatment of all cells studied.

Four different sets of conditions in each experiment were conducted for each cell line in conjunction with each chemotherapeutic agent: untreated control cells, cells treated by the chemotherapeutic agent alone, cells exposed to TTFields, and cells having a combined TTFields - Chemo exposure (8 Petri dishes for each condition). After 72 h, the culture media was discharged, XTT reagent was added and the final number of viable cells, OD_72 h_, was determined. Data obtained from 3 - 5 experiments were collected and the mean values and standard deviations (SEM) of OD_72 h_, representing final viable cell numbers were calculated for each set of conditions. Cell survival was presented as percentage of viable cells out of the corresponding viable cell number in untreated controls. Student t-test was applied to asses the significance of the differences between results obtained for each of the four conditions tested. In order to assess the extent of possible chemotherapeutic dose reduction when applied in combination with TTFields, dose reduction indexes (DRI) for each TTFields/drug combination were calculated according to [17].

The DRI for the same level of effect (DRI_m_) was calculated as the ratio of the concentration of drug alone to that of the combined drug-TTFields treatment:

DRI_m _= D_m(drug alone)_/D_m(combined treatment)_. The DRIs determine the magnitude of dose reduction allowed for each drug when given in combination with TTFields, as compared with the agent dose that achieves the same level of effect. DRI values larger than 1 indicate increased sensitivity to the drug.

### Intracellular Doxorubicin Accumulation

The intracellular accumulation of doxorubicin was determined for both wild type and drug resistant sub-lines. Cells were grown in total 16 Petri dishes (35 mm, NUNC) as monolayers for 24 h in drug-free medium and then incubated for 1 h in the absence or presence of doxorubicin with or without exposure to TTFields (1.75 V/cm, 150 kHz) (4 Petri dishes for each treatment condition). The cells were washed with ice cold PBS three times and solubilised with 100 μl of 2% SDS. The solutions were then transferred to black 96-well plates (NUNC) and doxorubicin fluorescence was measured by spectrofluorometry (ELISA Reader TECAN F-200) at λ_em _600 nm and λ_ex _450 nm. Data obtained from 2 - 4 experiments were collected and the mean values and standard deviations (SEM) of doxorubicin fluorescence were calculated for each condition. Student t-test was applied to asses the significance of the differences between results obtained for each of the three cell pairs.

## Results

### Effect of TTFields on wild type cells and their MDR sub-lines

**Figure 1:**
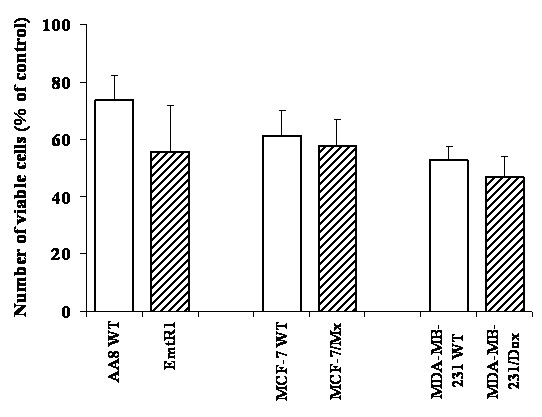
**The reduction in the number of viable WT and MDR cells following a 72 h exposure to TTFields**. Open bars - WT cells; filled bars - MDR cell sub-lines. TTFields intensity - 1.75 V/cm. Data presented as mean ± SEM of 30-36 replicate measurements from 4-5 experiments. Note that there is no statistical difference between WT and MDR pairs (student t-test).

In order to study the TTFields effect, field intensities that reduce the WT cell survival by about 50% were used. A comparison between the survival of wild type and MDR cells, when exposed to such TTFields, is given in Figure 1. The reduction in the number of viable cells is seen to be very similar (48-61% of control) in all wild type and paired MDR lines. In other words, the drug resistant cell lines have about the same sensitivity to TTFields as their corresponding wild type cell lines.

### Exposure to doxorubicin or paclitaxel in combination with TTFields

**Figure 2:**
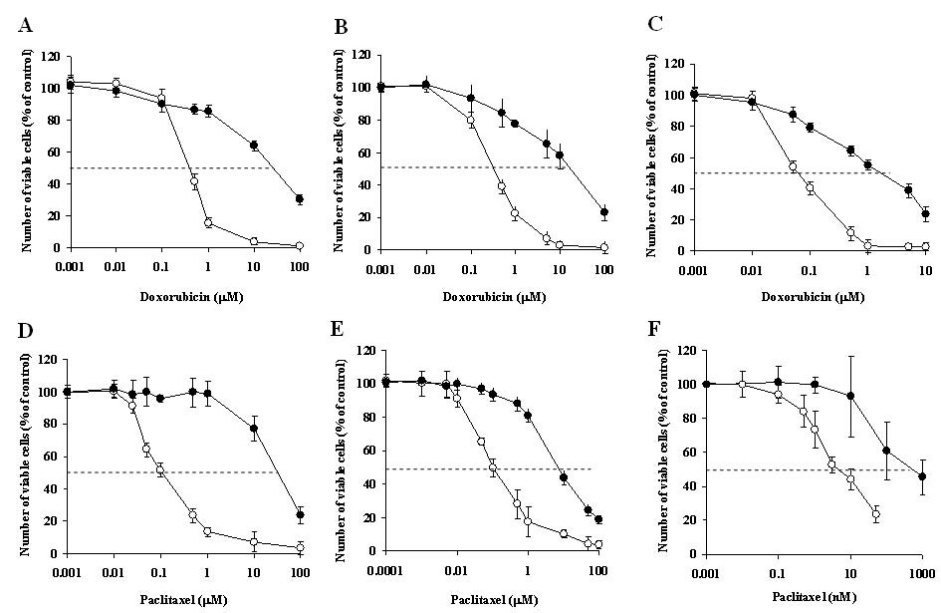
**Cytotoxicity of doxorubicin and of paclitaxel for wild type cells and the corresponding MDR sub-line cells**. A, B & C - doxorubicin. D, E & F - paclitaxel. A & D - AA8 & Emt^R1 ^cell lines; B & E - MCF-7 & MCF-7/Mx cell lines; C & F - MDA-MB-231 & MDA-MB-231/Dox cell lines. Open symbols -wild type cell lines. Filled symbols - MDR cell sub-lines. Treatment duration - 72 h. Data presented as mean ± SEM of 12-20 replicate measurements from 3-5 experiments.

Figure 2 compares between the cytotoxicity-dose curves of chemotherapeutic agents (paclitaxel and doxorubicin) of wild type cells and MDR sub-lines. It is seen that the resistivity of the MDR sub-lines is manifested in a significant right shift of the drug cytotoxicity-dose curves. As a result of these shifts the calculated IC_50 _values (Table 1) for doxorubicin and paclitaxel, for all pairs of WT-MDR cell lines studied, give very high IC_50 _ratios (resistance index RI): 55 - 79 for doxorubicin and 128 - 653 for paclitaxel.

**Table 1:** IC_50 _values for doxorubicin and paclitaxel

	IC_50_					
	
Drug	AA8	Emt^R1^	MCF-7	MCF-7/Mx	MDA-MB-231	MDA-MB-231/Dox
Doxorubicin (μM)	0.6	48.4	0.5	30.5	0.04	2.2
Paclitaxel (μM)	0.1	65.3	0.09	9.9	0.005	0.829

Drug concentrations inhibiting cell growth by 50% (IC_50_) were calculated from relative survival curves (see Figure 2) using the median-effect principle [16].

**Figure 3:**
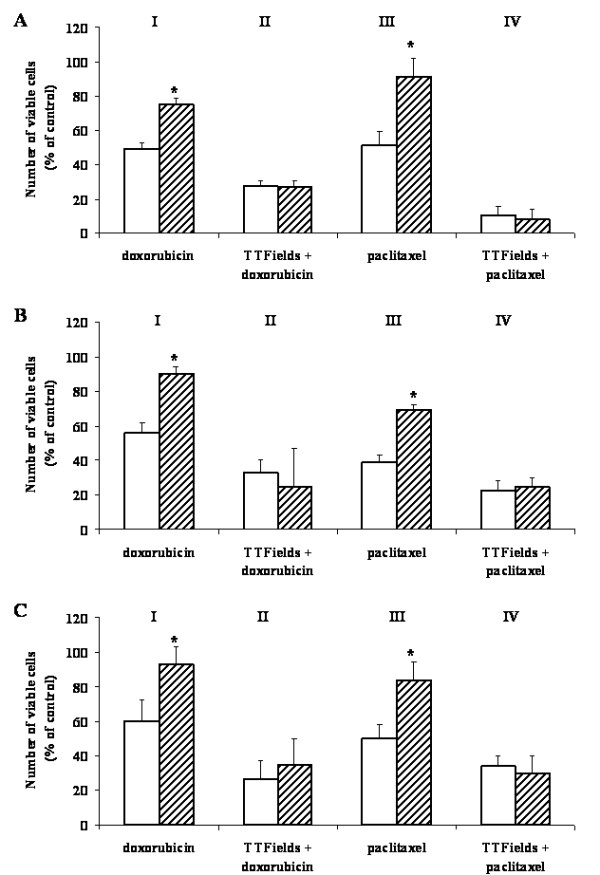
**Effects of doxorubicin and paclitaxel when applied separately and in combination with TTFields on the viability of wild type and MDR cells**. A - MDA-MB-231 & MDA-MB-231/Dox; B - MCF-7 & MCF-7/Mx; C - AA8 & Emt^R1^. Open bars - wild type cells; filled bars - MDR cell sub-lines. I & III **- **Separate exposures, II & IV - combined exposures. TTFields intensity - 1.75 V/cm. Doxorubicin concentrations: A - 0.04 μM; B - 0.5 μM; C - 0.6 μM. Paclitaxel concentrations: A - 5 nM; B -0.1 μM; C - 0.1 μM. Treatment duration - 72 h. Data presented as mean ± SEM of 24-36 replicate measurements from 3-5 experiments. * P < 0.01, student t-test.

A comparison between cell viability following separate and combined TTFields/drug exposures are presented in Figure 3. It is seen that in all combined exposures cell survival is lower as compared with exposure to any of the chemical agents (doxorubicin or paclitaxel) or TTFields alone (see Figure 1). Moreover, the cell survival of the MDR sub-lines and WT cell lines, when subjected to the combined exposure is similar, i.e. the resistivity or reduced drug sensitivity of MDR cells are not evident under these conditions.

Table 2 summarizes the combined treatment efficacy for MDR cells (see Figures 2 &3) expressed in terms of Dose Reduction Index (DRI). TTFields are seen to increase the sensitivity to doxorubicin of all three MDR sub-lines by at least two orders of magnitude. The corresponding increase for paclitaxel is even greater, i.e. two to three orders of magnitude. In other words, the efficacy of combined drug/TTFields treatment of MDR cells greatly exceeds that of treatment with drug alone.

**Table 2:** Dose reduction indexes for MDR cell sub-lines treated alone and in combination with TTFields.

	Dose reduction index (DRI)		
	
Drug	Emt^R1^	MCF-7/Mx	MDA-MB-231/Dox
Doxorubicin	105	195	250
Paclitaxel	815	4404	> 10,000

The DRI estimates the extent to which the dose of one or more agents in the combination can be reduced to achieve effect levels that are comparable with those achieved with single agents. The effect of TTFields/drug combined treatment for each MDR cell sub-line was as shown in Figure 3. The same effect of single drug was obtained from dose-response curves (see Figure 2). The DRI was calculated as a ratio of drug concentrations used alone vs. drug concentrations used in combination with TTFields.

### Intracellular Doxorubicin Accumulation

**Figure 4:**
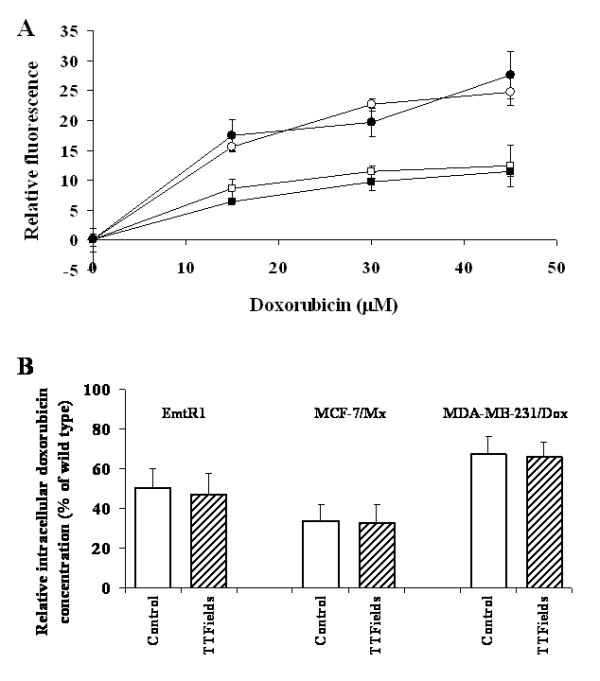
**Effect of TTFields on doxorubicin accumulation**. A - Dose response curve for AA8 cells and for their MDR sub-line Emt^R1^. Open symbols - cells exposed to drug alone; filled symbols - cells exposed simultaneously to drug and TTFields. Circles - AA8 cell line; squares - Emt^R1 ^sub line. Intensity of TTFields - 1.75 V/cm, frequency - 150 kHz. Treatment duration - 1 h. Data presented as means ± SEM of 16-24 replicate measurements from 2-3 experiments. B - Effect of TTFields on doxorubicin accumulation by different MDR cell sub-lines relative to their parental wild type cell lines. Ordinate: relative intracellular doxorubicin concentration in the drug resistant sub lines presented as % of the corresponding concentration in the wild type cells. Open bars - cells exposed to drug alone; filled bars - cells exposed simultaneously to drug and TTFields. Doxorubicin concentration: 30 μM. TTFields intensity - 1.75 V/cm, TTFields frequency - 150 kHz. Treatment duration - 1 h. Data are presented as mean ± SEM of 12-24 replicate measurements from 3-4 experiments.

An inherent feature of overexpressed ABC transporters phenotype is the reduction in cell uptake of doxorubicin due to its exclusion [18]. The ability of MDR cells to exclude doxorubicin was determined by means of spectrofluorometric analysis. Figure 4A illustrates the intracellular concentration of doxorubicin in AA8 (WT) and Emt^R1 ^(MDR) cell lines as a function of extracellular doxorubicin concentration with and without exposure to TTFields. As the drug is partially excluded from drug resistant sub line, the relative intracellular doxorubicin concentration in Emt^R1 ^cells is lower by 44.9, 49.7 and 49.8% at 15, 30 and 45 μM extracellular doxorubicin concentration respectively, as compared with the wild type cells (Figure 4A, open symbols). Exposure of AA8 (WT) and Emt^R1 ^(MDR) cell lines to TTFields during incubation with doxorubicin had no effect on the intracellular concentration of the drug in both wild type and drug resistant sub lines indicating that TTFields affect neither doxorubicin uptake nor its exclusion (Figure 4A, filled symbols). Figure 4B depicts doxorubicin accumulation by MDR sub lines relative to the corresponding WT cell lines exposed to 30 μM of doxorubicin with and without TTFields. The relative intracellular doxorubicin concentration is lower by 49.7 ± 5% for Emt^R1^, 66.4 ± 5% for MCF-7/Mx and by 32.6 ± 5% for MDA-MB-231/Dox as compared with the corresponding wild type cells (Figure 4B, open bars). TTFields have no effect on intracellular doxorubicin concentrations in all wild type and drug resistant cell lines (Figure 4B, filled bars).

## Discussion

ABC transporters provide vital protection from foreign compounds by exporting these compounds from the cell, thus lowering their intracellular concentration. Unfortunately, exposure of cancer cells to chemotherapeutics, mainly during relapse treatment, causes transporter upregulation such that the resulting over-expression of ABC transporters becomes one of the main causes of treatment failure. Moreover, various tumors such as renal cell, adrenocortical, colon and hepatocellular cancers express ABCB1 and are practically chemoresistant [19]. To overcome this problem chemosensitizers that block ABC transporter-mediated efflux were developed and have been used to combat MDR. However, this approach has not been clinically successful and therefore novel approaches that bypass, rather than block ABC transporters, are being sought for [20]. As the TTFields do not affect drug transport (see Figure 4) they fall into this category.

**Figure 5:**
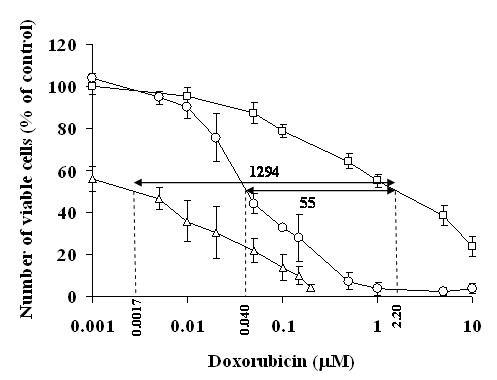
**Effect of 72 h application of TTFields and chemotherapeutic agents, separately and in combination on the viability of MDA-MB-231 wild type cells and MDA-MB-231/Dox MDR cells**. **-○- **MDA-MB-231 cells treated with doxorubicin alone; - △ - MDA-MB-231 cells treated with doxorubicin in combination with TTFields (ref. [9]); - □ - MDA-MB-231/Dox cells treated with doxorubicin alone.

The results of this study clearly indicate that both the MDR and WT cells are similarly sensitive to TTFields. Moreover, TTFields were shown to enhance MDR cell sensitivity to chemotherapeutic agents, so as to equal that of WT cells under the same set of conditions (Figure 3). This phenomenon can only be partially explained on the basis of the corresponding dose - response curves (Figure 2) and the drug export rate (Figure 4). As demonstrated in Figure 5, the dose - response curve of the drug resistant cells is shifted to the right relative to the WT cells (see also Figure 2). The magnitude of the shift is such that the 50% inhibition of WT cells that is obtained at a concentration of 0.04 μM requires a concentration of 2.2 μM for the MDR sub-line, i.e. a 55 fold higher concentration. However, the data depicted in Figure 4 and corresponding reports for low doxorubicin doses [21] indicate that the drug export lowers the intracellular concentration only by a factor of about 2. This means that some other factors must be responsible for the MDR resistance that corresponds to additional 20-30 fold drug concentration change. From the data in Figure 3A we also learn that both the MDR and WT cells are similarly highly sensitive to combined chemotherapy - TTFields treatments. Thus, while a 50% inhibition of MDR cells by doxorubicin alone requires a concentration of 2.2 μM, the combined treatment of TTFields and low concentration of doxorubicin (0.0017 μM) is sufficient to induce a similar inhibition. This is equivalent to an increased intracellular concentration of doxorubicin by a factor of over 1000. Thus, TTFields seem to have effects specific to MDR cells, not related to drug transport, that increase the MDR cell's sensitivity to chemotherapy. This conclusion is consistent with that of others [22-24] that attribute the MDR resistance, in addition to reduced drug uptake, to a number of potential mechanisms such as: sugar metabolism and energy production, alterations in cytoskeletal elements, microtubule and mitochondria distribution, etc. Within the framework of the above suggested mechanisms [22-24] it seems that the integrity of cytoskeleton and microtubule as well as the mitochondria distribution may be the most vulnerable to the forces produced by TTFields. The former may be disrupted by particle movements induced by the dielectrophoresis induced during TTFields application [8] while the latter are highly polar in themselves and are therefore directly subjected to the alternating field forces.

## Conclusions

The results of this study support the notion that TTFields may be used, both as an effective stand alone anti-proliferation agent for MDR cells, as well as an effective adjuvant that enhances chemotherapy efficacy. Furthermore, since TTFields are a physical modality, their therapeutic efficacy is independent of interaction with cell receptors. Therefore their efficacy is not expected to be limited to a specific set of cell types [8-12]. On the basis of the above, we believe that there is a high probability that TTFields may prove to be an effective therapeutic modality to a wide range of human cancers including those that developed multi drug resistance.

## List of abbreviations

MDR: multidrug resistance; TTFields: tumor treating electric fields; DRI: dose reduction index; WT: wild type.

## Competing interests

RSS, ES and EK are employees of NovoCure Ltd. YP has a minority holding in NovoCure Ltd.

## Authors' contributions

YP - Conceived the concept of TTFields, designed experiments, was involved in data analysis & interpretation of results and wrote the majority of the manuscript. RSS - Participated in experimental design, supervised the experiment execution, analyzed results and wrote parts of the manuscript. ES - Carried out the experiments. EDK - Participated in experimental design and in the interpretation of the results.

All authors read and approved the final manuscript.

## Pre-publication history

The pre-publication history for this paper can be accessed here:

http://www.biomedcentral.com/1471-2407/10/229/prepub
